# Intravenous anesthetic propofol suppresses T cell–dependent antibody production in mice

**DOI:** 10.1007/s00540-025-03533-7

**Published:** 2025-06-20

**Authors:** Susumu Hiraoka, Hiroki Satooka, Hirotoshi Kitagawa, Takako Hirata

**Affiliations:** 1 Department of Fundamental Biosciences, Shiga University of Medical Science, Seta-Tsukinowa-Cho, Otsu, Shiga 520-2192 Japan; 2 Department of Anesthesia, Shiga University of Medical Science, Otsu, Shiga 520-2192 Japan

**Keywords:** Propofol, Immune response, Antibody production, T cells

## Abstract

**Purpose:**

General anesthesia combined with surgery is thought to suppress the immune system. However, few studies have examined the effects of anesthetics alone on humoral immunity. In this study, we aimed to investigate the effects of the intravenous anesthetic propofol on antibody production after immunization and the underlying mechanisms in mice.

**Methods:**

Mice were immunized with 4-hydroxy-3-nitrophenylacetyl (NP) hapten–conjugated keyhole limpet hemocyanin (NP–KLH), a T cell–dependent antigen, or NP hapten–conjugated Ficoll (NP–Ficoll), a T cell–independent antigen, followed by treatment with propofol, or PBS or intralipid as controls, for five consecutive days. The mice were re-immunized, and antibody production and immune cell subsets were evaluated. The effects of propofol on T cell proliferation and survival were also examined.

**Results:**

NP-specific IgM and IgG1 titers were reduced in propofol-treated NP–KLH–immunized mice compared to those treated with PBS or intralipid, and this reduction was more pronounced in the secondary response than in the primary response. By contrast, propofol treatment did not affect NP-specific antibody titers in NP–Ficoll–immunized mice. In vitro, propofol inhibited IL-2–mediated proliferation and IL-7–dependent survival of CD4^+^ T cells.

**Conclusions:**

Propofol suppresses T cell–dependent antibody production in mice and directly affects T cell proliferation and survival in vitro. These data suggest that anesthetics administered close to the time of vaccination may affect vaccine-specific antibody production.

**Supplementary Information:**

The online version contains supplementary material available at 10.1007/s00540-025-03533-7.

## Introduction

General anesthesia during surgery generally suppresses the immune system and may affect postoperative complications and cancer recurrence. It may also have an influence on the efficacy and safety of perioperative vaccinations. During the recent COVID-19 pandemic, many patients have received vaccinations against novel coronavirus SARS-CoV-2 in the pre- and postoperative periods, and anesthesia may have affected immune response against the vaccine [1]. However, studies on the effect of anesthetics on the immune response after vaccination are limited. In particular, the effects of anesthetics alone on antibody production after immunization have largely been unexamined.

Many studies have reported the effects of both inhalation and intravenous anesthetics on the innate and acquired immune system [2]. Propofol is an intravenous anesthetic widely used for the induction and maintenance of anesthesia and for sedation in various medical procedures. Repeated treatment of mice with propofol reduces CD4^+^ T cell frequency in the peripheral blood mononuclear cells [3] and suppresses T cell function and T cell–dependent immune responses [4]. By contrast, in patients undergoing craniotomy, propofol anesthesia alleviates the surgical stress–induced decrease in the T helper 1/T helper 2 ratio [5], which is associated with suppression of cell-mediated immunity, suggesting that propofol may not affect cell-mediated immunity, but its effect on humoral immunity remains largely obscure.

Many anesthetics act through potentiating GABA type A (GABA_A_) receptors, the most abundant inhibitory neurotransmitter receptors in the central nervous system. GABA_A_ receptors are ligand-gated ion channels that inhibit neuronal activity by allowing chloride ions (Cl^−^) to flow into the cell. Propofol binds to multiple sites on GABA_A_ receptors and enhances GABA-induced Cl^−^ currents [6, 7]. GABA_A_ receptors are formed as pentamers composed of two α-subunits (α1–6), two β-subunits (β1–3), and one γ-subunit (γ1–3, δ, ε, θ, π, ρ) in various combinations of subunits. It is becoming clear that GABA_A_ receptors are also expressed in cells other than neurons, including immune cells. Indeed, human monocytes and T and B cells express certain sets of subunits that can form functional GABA_A_ receptors [8, 9], implicating that anesthetics acting on GABA_A_ receptors may directly affect immune cells.

In this study, we report that repeated treatment of mice with propofol suppresses the production of antibodies against 4-hydroxy-3-nitrophenylacetyl (NP) hapten–conjugated keyhole limpet hemocyanin (NP–KLH), a T cell–dependent antigen. By contrast, propofol treatment had little effect on the production of antibodies against NP hapten–conjugated Ficoll (NP–Ficoll), a T cell–independent antigen. In vitro, propofol directly suppressed the IL-2–mediated proliferation and IL-7–dependent survival of CD4^+^ T cells. Collectively, our results suggest that propofol suppresses antibody production through T cell–dependent processes, likely through its direct effect on T cells.

## Methods

### Mice

Female C57BL/6 mice were purchased from Japan SLC (Hamamatsu, Japan) and housed at the Research Center for Animal Life Science at Shiga University of Medical Science. Mice were used at eight to 10 weeks of age. All experiments were approved by the Animal Research Committee of Shiga University of Medical Science (Approval No. 2021-11-12(H2)).

### Immunization

Mice were immunized intraperitoneally with 100 μg NP–KLH (NP:KLH, 24:1; NP_24_–KLH; Biosearch Technologies, Teddington, UK) or NP–Ficoll (NP:aminoethyl carboxymethyl (AECM)–Ficoll, 54:1; NP_54_–AECM–Ficoll; Biosearch Technologies) precipitated in alum (Imject Alum; Thermo Fisher Scientific, Waltham, MA) on day 0. The mice were re-immunized intraperitoneally with 100 µg NP–KLH or NP–Ficoll in PBS on day 49. Sera were collected on days 0, 4, 7, 14, 21, 28, 35, 42, 49, 56, and 63.

### Propofol anesthesia in mice

The mice were intravenously injected with a bolus of 26 mg/kg propofol (2,6-diisopropylphenol; 1% Diprivan Injection; Sandoz Pharma, Basel, Switzerland) through the tail vein. This dose induced loss of righting reflex (LORR), which has been used in animals to assess loss of consciousness. Since propofol is formulated as an oil-in-water emulsion containing 100 mg/ml soybean oil, 22.5 mg/ml glycerol, and 12 mg/ml purified egg phospholipids, the control groups received intralipid (10% Intralipos Injection; Otsuka Pharmaceutical, Tokyo, Japan), which has a similar lipid content (100 mg/ml soybean oil, 22 mg/ml glycerol, and 12 mg/ml purified egg phospholipids), or PBS. While the mice were sleeping, the rectal temperature was maintained at 37°C or higher using a temperature maintenance system for small animals (BWT-100A; Bio Research Center, Nagoya, Japan). The mice were monitored while sleeping and the duration of LORR was recorded. Propofol was administered once daily for five consecutive days from day 0 to day 4, following a published protocol [3]. For immunized mice, administration on day 0 was performed 4 h after immunization. The immunization and anesthesia schedules are shown in Supplementary Fig. 1.

### ELISA

Total and NP-specific antibody titers were determined using sandwich ELISA as described previously [10]. For total antibody titers, the plates were coated with goat anti-mouse IgM, IgG1, or IgG3 (0.25 µg/well; Bethyl Laboratories, Montgomery, TX) and blocked with 0.2% BSA in PBS, followed by the addition of serially diluted serum samples and standards. Serum antibodies were detected using horseradish peroxidase (HRP)–conjugated goat antibodies against each isotype (Bethyl Laboratories). TMB (3,3′,5,5′-tetramethylbenzidine; Thermo Fisher Scientific) was added as a substrate and the reaction was stopped with 2M H_2_SO_4_. Absorbance was measured at 450 nm using an Infinite 200 PRO microplate reader (Tecan, Männedorf, Switzerland).

For NP-specific antibody titers, the plates were coated with NP_4_–BSA or NP_52_–BSA (0.25 µg/well; Biosearch Technologies) and blocked with 0.2% BSA in PBS, and then serially diluted serum samples were added. The NP-specific IgM, IgG1, and IgG3 antibodies were detected using HRP-conjugated goat antibodies against each isotype. The plates were developed as described above. Antibody titers were determined using reference serum.

### Cell preparation

Lymphocytes were isolated from spleens by mechanical disruption between the frosted surface of two glass slides, followed by filtration through 100-μm nylon mesh twice. The collected cells were quantified using a hemocytometer.

For T cell proliferation and survival assays, naive CD4^+^ T cells were isolated from splenocytes with MACS using a Naive CD4^+^ T Cell Isolation Kit (Miltenyi Biotec, Bergisch Gladbach, Germany).

### Flow cytometry

The monoclonal antibodies used for flow cytometric analyses were purchased from BD Biosciences (San Jose, CA), eBioscience (San Diego, CA), BioLegend (San Diego, CA), or Miltenyi Biotec and included those to B220 (RA3-6B2), CD3 (145-2C11), CD4 (RM4-5), CD8 (53-6.7), CD19 (6D5), CD25 (PC61.5), CD38 (90), CD44 (IM7), CD45RB (C363-16A), CXCR-5 (RF8B2), FAS (Jo2), Foxp3 (FJK-16s), GL-7 (GL-7), IgD (11-26c.2a), and PD-1 (RPM1-30). Single-cell suspensions were incubated with anti-CD16/CD32 (93) for 10 min, stained with antibodies for 30 min on ice, and washed. For intracellular staining of Foxp3, surface-stained cells were fixed and permeabilized using a Foxp3/Transcription Factor Staining Buffer Kit (eBioscience). Data were acquired on an LSRFortessa X-20 (BD Biosciences) and analyzed using FlowJo. The gating strategy is shown in Supplementary Fig. 2.

### T cell proliferation and survival assays

For T cell proliferation assays, the isolated cells were stained with carboxyfluorescein succinimidyl ester (CFSE; CellTrace CFSE Cell Proliferation Kit, for flow cytometry; Thermo Fisher Scientific) following the manufacturer’s instructions. The stained cells were cultured in plates precoated with 10 µg/ml anti-CD3 in complete RPMI 1640 containing 10 µg/ml anti-CD28, with or without 20 ng/ml IL-2, 50 µM GABA (TCI, Tokyo, Japan), and 2,6-diisopropylphenol (TCI). After 72 h of incubation, the cells were analyzed by flow cytometry.

For T cell survival assays, the isolated cells were cultured in complete RPMI 1640 containing 20 ng/ml IL-2, IL-7, or IL-15 for 48 h in 96-well plates, stained with DAPI, and analyzed by flow cytometry. To assess apoptosis, the cells were stained with Annexin V (BD Biosciences) and propidium iodide (PI).

### Statistical analysis

Data are represented as the mean ± SEM, and statistical analysis was performed using Prism 9 (GraphPad Software, San Diego, CA). Differences between groups were analyzed using one-way or two-way ANOVA, and Tukey’s and Dunnett’s multiple comparison tests were performed. Statistical significance was defined as *p* < 0.05.

## Results

### Propofol treatment suppresses antibody production

**Fig. 1:**
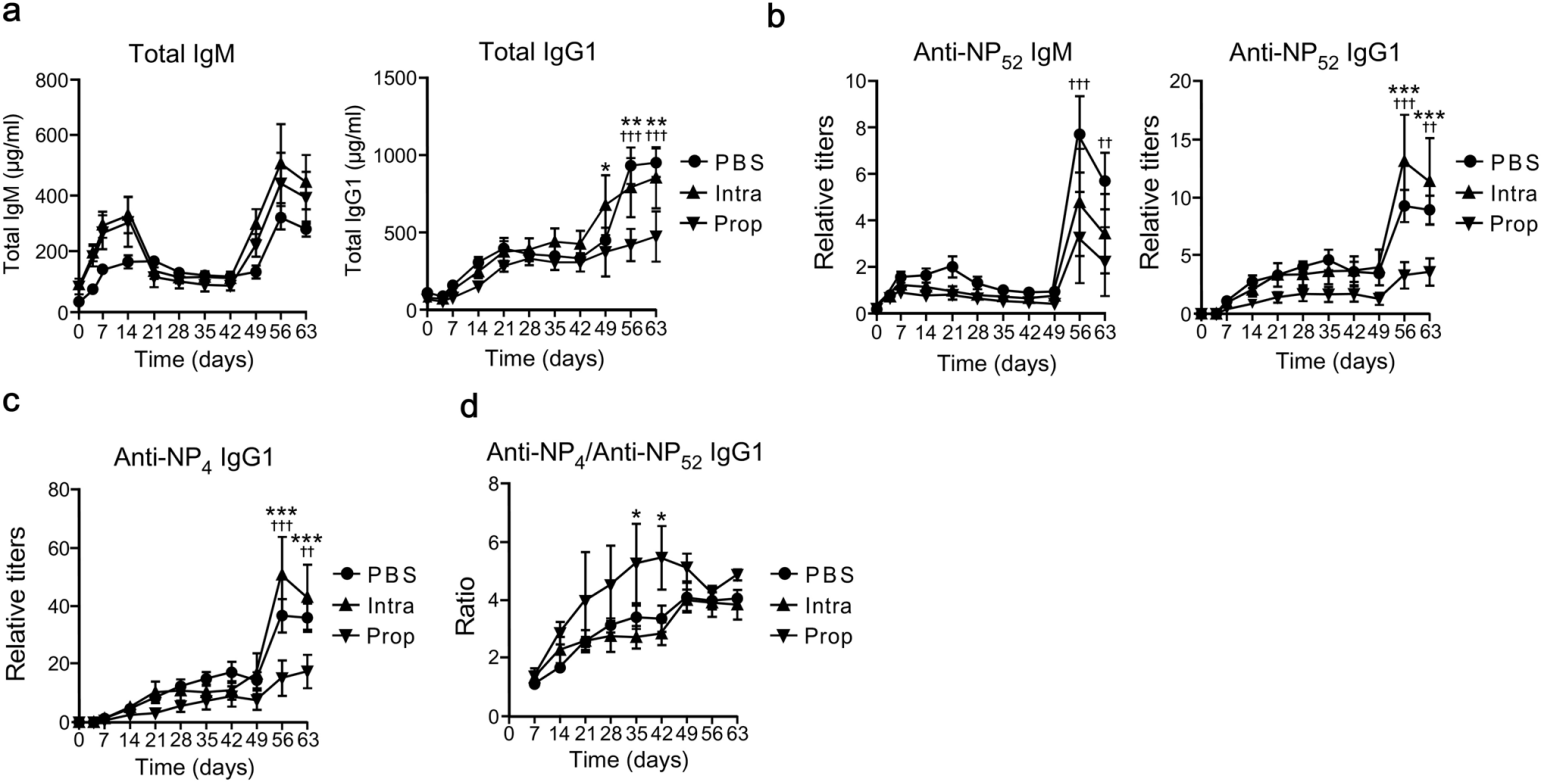
Effects of propofol on T cell–dependent antibody production. **a**–**c** Total IgM and IgG1 titers (**a**), total NP-specific IgM and IgG1 titers measured with NP_52_–BSA–coated plates (**b**), and high-affinity NP-specific IgG1 titers measured with NP_4_–BSA–coated plates (**c**) in the serum of NP–KLH–immunized mice treated with PBS, intralipid (Intra), or propofol (Prop). **d** The ratios of high-affinity anti-NP_4_ IgG1 to total anti-NP_52_ IgG1. Data are presented as mean ± SEM (*n* = 6 mice per group). Intralipid vs. propofol, *p < 0.05, **p < 0.01, ***p < 0.001; PBS vs. propofol, ^††^*p* < 0.01, ^†††^*p* < 0.001

To investigate the effect of propofol on humoral immunity, we examined serum antibody titers in mice after immunization with NP–KLH, a T cell–dependent antigen, on day 0 and booster on day 49. The immunized mice were treated with propofol once a day for five consecutive days, with the first dose administered 4 h after immunization on day 0. The control mice were administered PBS or intralipid, which has a similar lipid content as the propofol formulation used in this study. Propofol-induced LORR duration was 375 ± 105 s, which was not significantly different from that for unimmunized mice (397 ± 97 s). Although total IgM levels in propofol-treated mice did not differ from those in PBS or intralipid-treated mice, total IgG1 levels were significantly lower than those in the PBS- or intralipid-treated mice in the secondary response (Fig. 1a). To evaluate antigen-specific responses, we examined NP-specific antibody titers. Total NP-specific IgM and IgG1 titers, measured with NP_52_–BSA–coated plates, tended to be low in the propofol-treated mice during the primary response and significantly reduced during the secondary response compared to PBS- or intralipid-treated mice (Fig. 1b). High-affinity NP-specific IgG1, measured with NP_4_–BSA–coated plates, also decreased in the propofol-treated mice during the secondary response (Fig. 1c). Although the ratio of high-affinity to total NP-specific IgG1 gradually increased after immunization in all groups, propofol-treated mice showed a slightly higher ratio in the primary response, which rather decreased during the secondary response (Fig. 1d). These results suggested that propofol treatment suppressed antibody production, with the effect being more apparent during the secondary response.

### Propofol alters T and B cell composition

**Fig. 2:**
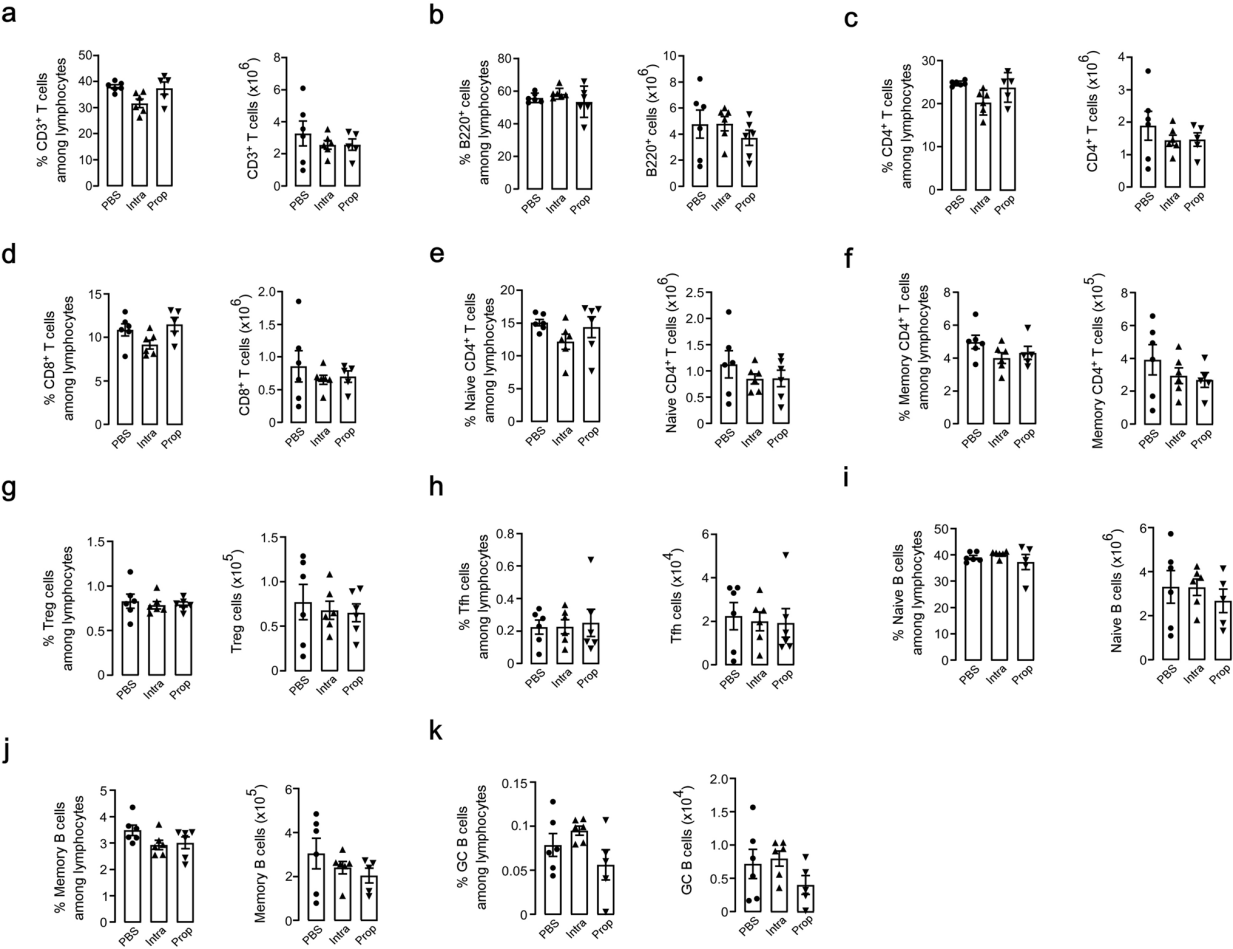
Effects of propofol on T and B cell subsets in the spleen of mice immunized with T cell–dependent antigen. The percentage and number of T cells (**a**), B cells (**b**), CD4^+^ T cells (**c**), CD8^+^ T cells (**d**), naive CD4^+^ T cells (**e**), memory CD4^+^ T cells (**f**), Treg cells (**g**), Tfh cells (**h**), naive B cells (**i**), memory B cells (**j**), and GC B cells (**k**) in the spleen isolated on day 63 from NP–KLH–immunized mice treated with PBS, intralipid, or propofol. Each subset was identified as shown in Supplementary Fig. 2. Data are presented as mean ± SEM (*n* = 6 mice per group), with each data point representing an individual mouse

As propofol treatment suppressed antigen-specific antibody production, we examined whether propofol affected immune cell subsets in the spleen. Flow cytometric analysis of the spleen on day 63 revealed that the percentage and number of T and B cells did not differ between the propofol- and intralipid or PBS-treated mice (Fig. 2a, b). The percentage and number of CD4^+^ T cells, both naive and memory, and CD8^+^ T cells were also comparable among the groups (Fig. 2c–f). Regulatory CD4^+^ T (Treg) cells, which exert suppressive control over other immune cells, were also similar among groups (Fig. 2g).

During T cell–dependent immune responses, antigens are transported to T cell zones and B cell follicles to initiate the activation of follicular helper T (Tfh) cells, a subpopulation of CD4^+^ T cells, and their interaction with B cells, resulting in germinal center (GC) reactions that produce high-affinity antibodies. Although the percentage and number of Tfh, naive B, memory B, and GC B cells varied in each group, GC B cells tended to be reduced in propofol-treated mice (Fig. 2h–k).

**Fig. 3:**
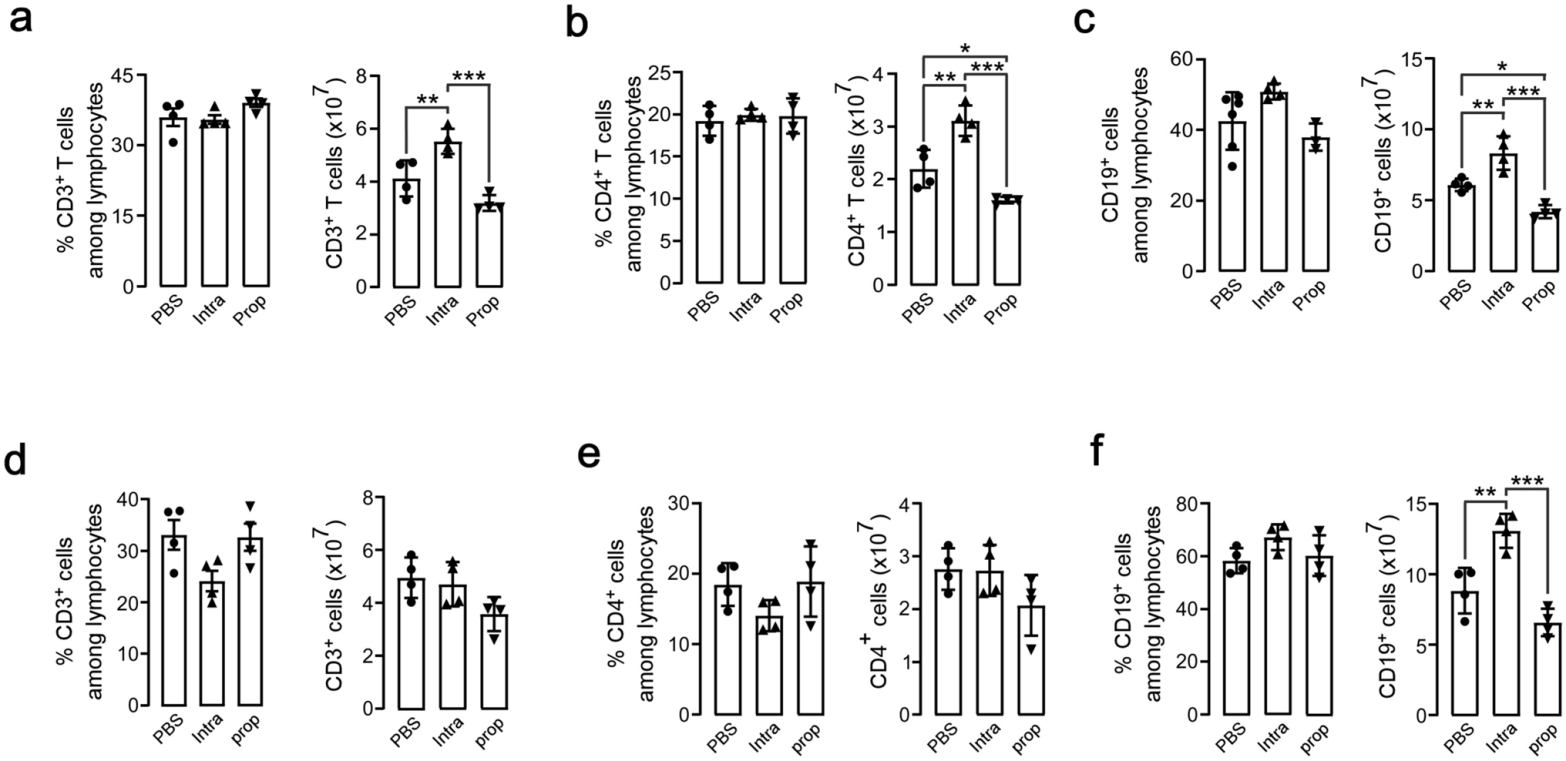
Short-term effects of propofol on T and B cell subsets in the spleen. The percentage and number of T cells (CD3^+^) (**a** and **d**), CD4^+^ T cells (CD3^+^CD4^+^) (**b** and **e**), and B cells (CD19^+^) (**c** and **f**) in the spleen isolated on day 5 from unimmunized mice (**a**–**c**) or NP–KLH–immunized mice (**d**–**f**) treated with PBS, intralipid, or propofol. Data are presented as mean ± SEM (*n* = 4 mice per group), with each data point representing an individual mouse. *p < 0.05, **p < 0.01, ***p < 0.001

To clarify the short-term influence of propofol on T and B cell composition, we examined the spleens of unimmunized and immunized mice on day 5, 24 h after the fifth propofol treatment. Flow cytometric analysis revealed that intralipid treatment increased the number of total T cells, CD4^+^ T cells, and CD19^+^ B cells in unimmunized mice, whereas propofol treatment decreased these numbers (Fig. 3a–c). In immunized mice, propofol decreased the T and B cell numbers, albeit to a lesser extent (Fig. 3d–f). These results suggest that lipid emulsion formulation and pharmacologically active components of propofol affect T and B cell homeostasis in opposite directions.

### Propofol suppresses T cell–dependent processes of antibody production

**Fig. 4:**
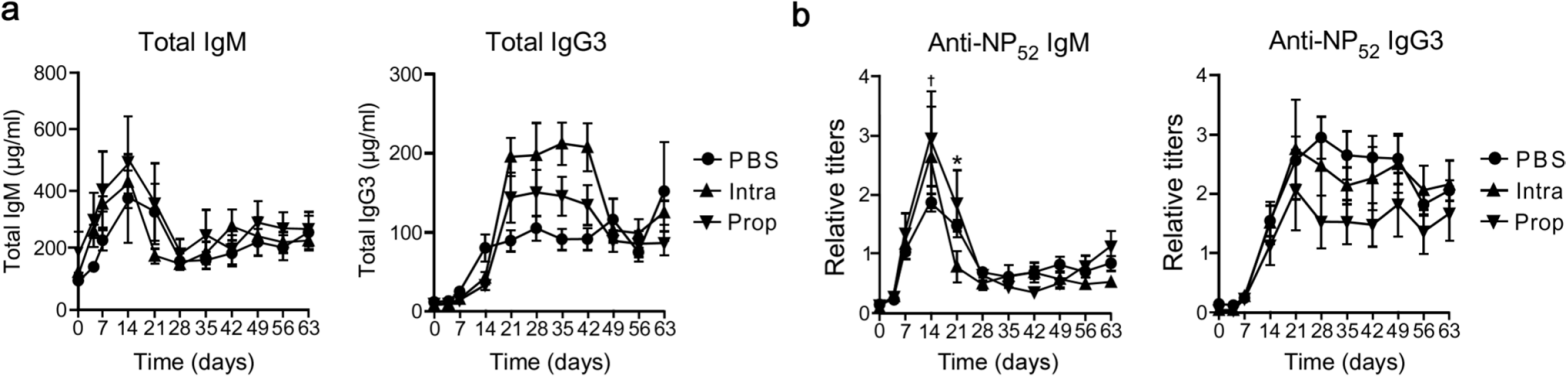
Effects of propofol on T cell–independent antibody production. Total IgM and IgG3 titers (**a**) and total NP-specific IgM and IgG3 titers measured with NP_52_–BSA–coated plates (**b**) in the serum of NP–Ficoll–immunized mice treated with PBS, intralipid, or propofol. Data are presented as mean ± SEM (*n* = 6 mice per group). Intralipid vs. propofol, *p < 0.05; PBS vs. propofol, ^†^*p* < 0.05

NP–KLH is a T cell–dependent antigen that induces complex cell–cell communication networks between antigen-reactive T and B cells and generates high-affinity IgG antibodies, predominantly the IgG1 subclass. By contrast, T cell–independent antigens mainly produce the low-affinity IgM and IgG3 subclass. To investigate whether propofol affects T cell–independent antibody production, we immunized mice with NP–Ficoll using the same immunization schedule as that used for NP–KLH. The total IgM and IgG3 levels were not significantly different among the three groups (Fig. 4a). NP-specific IgM in the propofol-treated group was rather high on days 14 and 21 compared to the PBS- or intralipid-treated groups, whereas NP-specific IgG3 titers were not significantly different among the groups (Fig. 4b). Flow cytometric analysis of the spleen revealed that propofol exerted a minor effect on the percentage and number of T and B cell subsets, including GC B cells, in NP–Ficoll–immunized mice (Supplementary Fig. 3a–k). Taken together, these results suggest that propofol affects T cell–mediated processes of antibody production.

### Propofol inhibits IL-2–mediated T cell proliferation

As propofol suppresses T cell–dependent antibody production, we explored whether propofol acts directly on T and B cells. Because propofol exerts its effects primarily via GABA_A_ receptors, we examined their expression in T and B cells. Both mouse T and B cells expressed α2, α3, α5, β2, β3, γ1, γ2, δ, θ, π, and ρ1 subunits, albeit at low levels compared to the brain (Supplementary Fig. 4a, b), implicating the possibility of the presence of functional GABA_A_ receptors on both T and B cells.

**Fig. 5:**
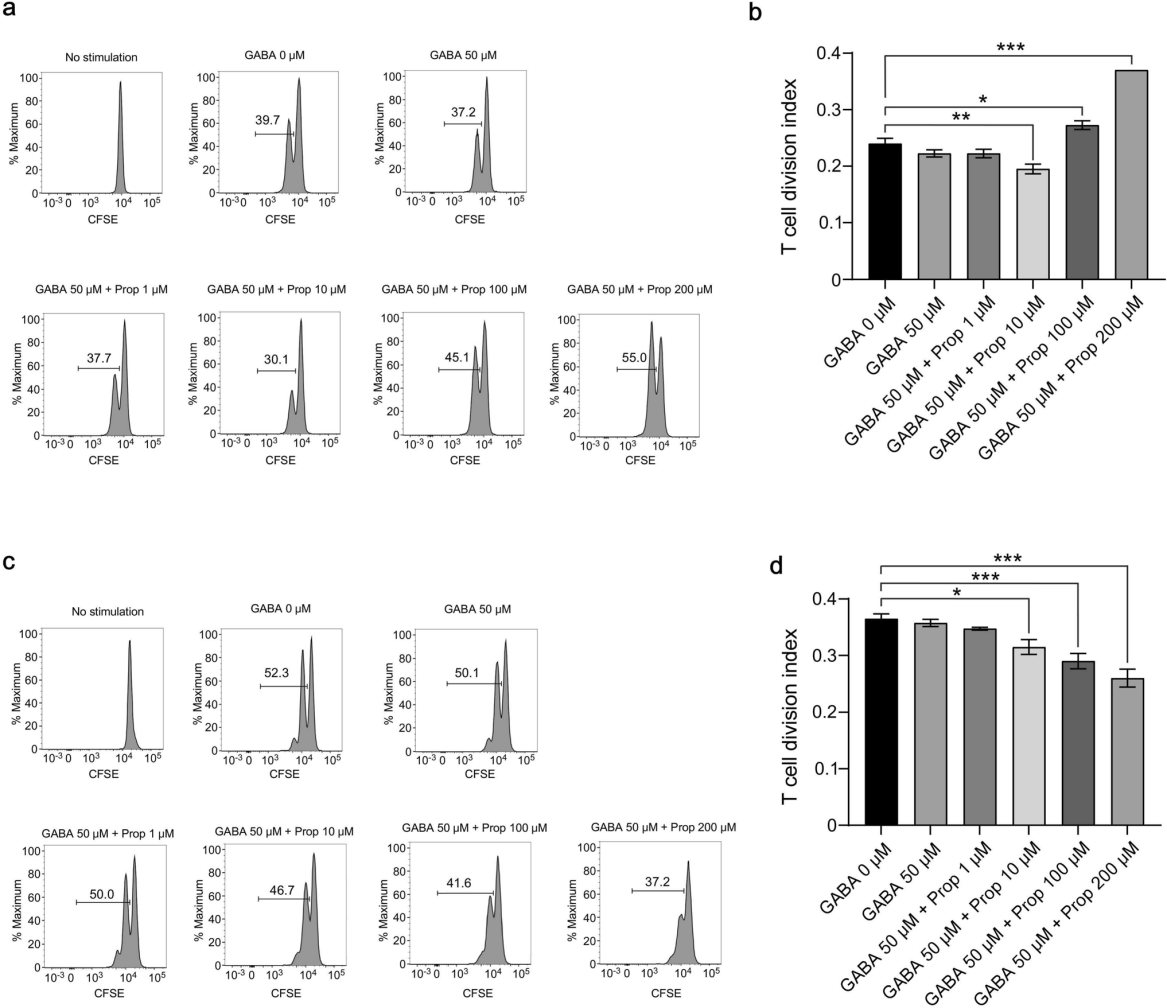
T cell proliferation in the presence of GABA and propofol. **a** and **b** Effects of GABA and propofol on TCR-stimulated T cell proliferation. Representative histograms (**a**) and T cell division index (**b**) of CFSE-labeled naive CD4^+^ T cells stimulated with anti-CD3 and anti-CD28 with or without GABA and propofol. **c** and **d** Effects of GABA and propofol on TCR- and IL-2–stimulated T cell proliferation. Representative histograms (**c**) and T cell division index (**d**) of CFSE-labeled naive CD4.^+^ T cells stimulated with anti-CD3, anti-CD28, and IL-2 with or without GABA and propofol are shown. Data are presented as mean ± SEM (*n* = 4 wells per group) and represent three independent experiments. *p < 0.05, **p < 0.01, ***p < 0.001

During T cell–dependent antibody production, presented antigens are recognized with T cell receptors (TCRs), allowing T cells to proliferate. Thus, we examined the effect of propofol on the TCR-stimulated proliferation of naive CD4^+^ T cells. Propofol at 10 μM in the presence of GABA slightly decreased proliferation but increasing propofol doses conversely increased proliferation (Fig. 5a, b). By contrast, when T cell proliferation was examined in the presence of exogenous IL-2, propofol dose-dependently inhibited proliferation (Fig. 5c, d). Under this assay condition, adding bicuculline, a GABA_A_ receptor antagonist, reversed proliferation inhibition by propofol (Supplementary Fig. 5a, b). B cell proliferation stimulated by LPS and anti-IgM was not suppressed but rather increased by propofol in a dose-dependent manner (Supplementary Fig. 6a, b). These results suggest that propofol directly suppresses T cell proliferation by acting on IL-2–mediated signaling.

### Propofol suppresses IL-7–dependent T cell survival

**Fig. 6:**
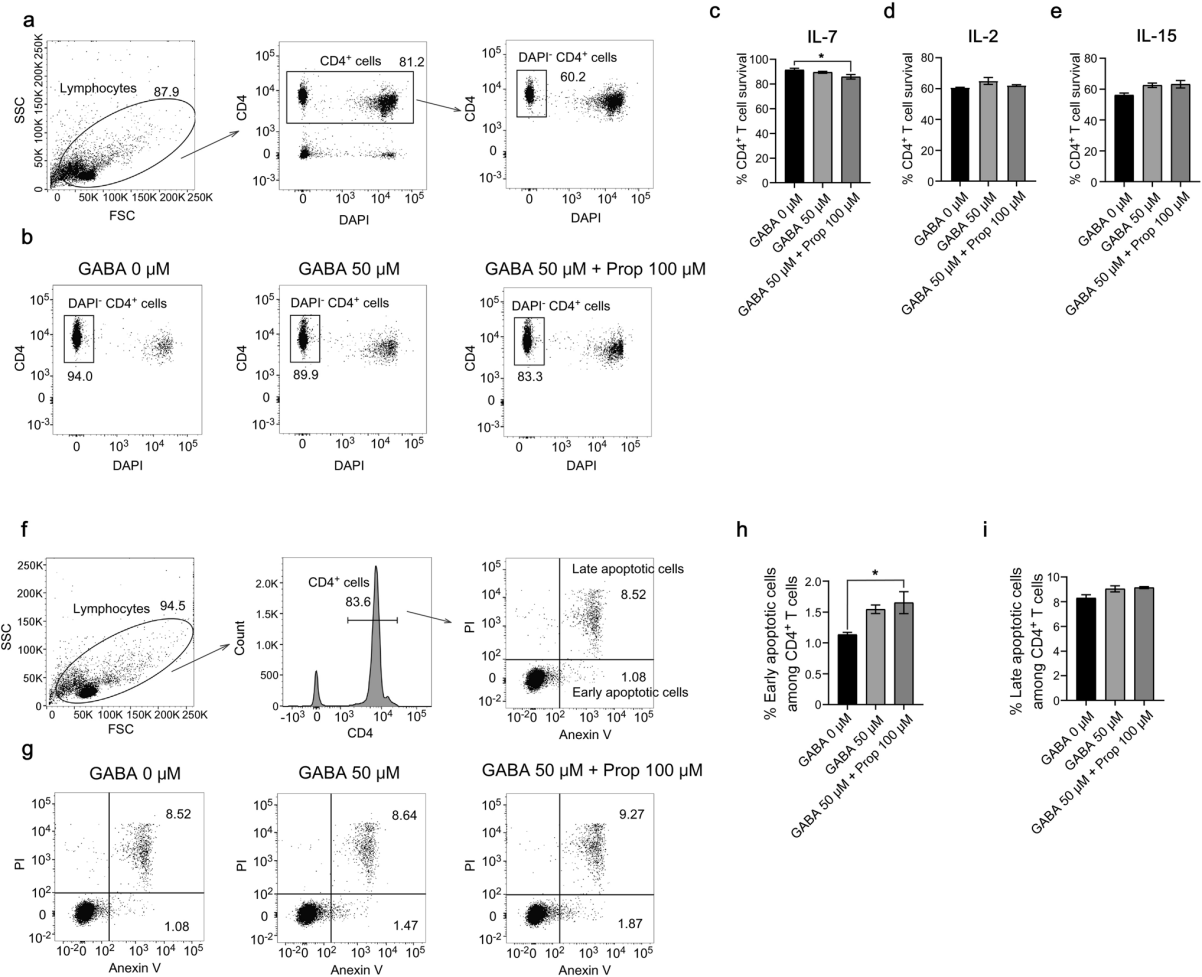
T cell survival in the presence of GABA and propofol. **a**–**e** Effects of GABA and propofol on T cell survival in the presence of IL-7, IL-2, or IL-15. Naive CD4^+^ T cells were cultured in the presence of indicated cytokines and viable CD4^+^ T cells were identified as DAPI^−^ cells. Gating strategy (**a**), representative plots in the presence of IL-7 (**b**), and the percentage of viable CD4^+^ T cells in the presence of IL-7 (**c**), IL-2 (**d**), or IL-15 (**e**) with or without GABA and propofol are shown. **f**–**i** Effects of GABA and propofol on T cell apoptosis in the presence of IL-7. Naive CD4^+^ T cells were cultured in the presence of IL-7 and early and late apoptotic cells were identified as Annexin V^+^PI^−^ and Annexin V^+^PI^+^, respectively. Gating strategy (**f**), representative plots (**g**), and the percentage of early (**h**) and late (**i**) apoptotic CD4^+^ T cells with or without GABA and propofol are shown. Data are presented as mean ± SEM (*n* = 3 wells per group) and represent three independent experiments. *p < 0.05

As propofol treatment in unimmunized mice decreased T cell numbers in the short term, we examined the effect of propofol on T cell survival. The survival of naive CD4^+^ T cells in the presence of IL-7, a cytokine critical for the homeostatic proliferation and survival of naive T cells [11], was significantly reduced with the addition of propofol (Fig. 6a–c). Contrastingly, propofol exerted no effect on cell survival in the presence of IL-2 or IL-15, which do not support naive T cell survival, as the cells do not express IL-2 receptor α-chain (CD25) and IL-15 receptor α-chain (CD215) (Fig. 6d, e). Because IL-7 supports naive T cell survival by inhibiting apoptosis, we stained the cells with Annexin V and PI after 48 h of culture (Fig. 6f). Treatment with propofol increased the percentage of early apoptotic cells more than that of late apoptotic cells (Fig. 6g–i), suggesting that propofol decreases T cell numbers by interfering with IL-7–mediated inhibition of apoptosis.

## Discussion

The effects of anesthesia combined with surgery on the immune system have been extensively studied; however, relatively few reports have investigated the effects of anesthesia alone on humoral immunity. In this study, we showed that propofol suppresses T cell–dependent antibody production in mice during the secondary response. In vitro, propofol interfered with IL-2–mediated T cell proliferation and IL-7–dependent T cell survival. These direct effects of propofol on T cells are likely responsible, at least partially, for suppressing antibody production.

Perioperative vaccination has been widely discussed, particularly during the recent COVID-19 pandemic, although few hospitals have set policies for the timing of vaccination with respect to anesthesia and surgery. The current general practice is to delay elective surgery by 2–7 and 14–21 days after administering inactivated and live vaccines, respectively [1, 12]. As propofol affects T cell proliferation and survival, which are required to maintain sufficient T cell numbers for optimal T cell responses, vaccination in close proximity to propofol anesthesia may be discouraged. Further studies are needed to determine the period required for the effect of propofol to be reduced for optimal vaccine-induced immune responses. Our observation that propofol treatment at the primary immunization caused a more pronounced suppression in antibody production in the secondary response than in the primary response may imply that propofol can affect the protective effect of vaccines against later infection.

Propofol is formulated as a lipid emulsion that causes formulation-related adverse reactions. The administration of lipid preparations induces an inflammatory response [13, 14]. Previous studies on heathy participants have shown that propofol anesthesia without any surgical intervention has both inflammatory and anti-inflammatory effects [15, 16], which may be attributed to the lipid formulation. In our study, intralipid administration to unimmunized mice increased both T and B cells in the spleen, while propofol administration led to their reduction, suggesting that the active component of propofol exerts its inhibitory effect, counteracting lipid-induced effects. To avoid lipid-induced effects, water-soluble propofol prodrugs have been developed [17], although lipid emulsion formulations are still widely used. Therefore, the effects of lipids should be considered while evaluating the effects of propofol on the immune system.

It is becoming increasingly clear that GABA plays a broader role beyond its actions in the central nervous system. GABA_A_ receptors are widely expressed in immune cells in both humans and mice [18]. In this study, we confirmed that mouse T and B cells express a similar set of GABA_A_ receptor subunits, suggesting the possibility that anesthetics targeting GABA_A_ receptors may act directly on T and B cells to exert inhibitory effects on acquired immunity. Consistent with this, benzodiazepines were reported to inhibit mouse and human T cell proliferation via GABA_A_ receptor activation [9]. GABA_A_ receptor overexpression also promotes apoptosis, which is blocked by the co-expression of Bcl-2, a molecule that regulates permeability of mitochondrial membrane to inhibit apoptosis [19]. Because IL-7–dependent T cell survival is also regulated by Bcl-2 expression, propofol may be involved in Bcl-2–mediated regulation of apoptosis. Our study also showed that propofol suppressed IL-2–mediated T cell proliferation. Given that IL-2 and IL-7 receptors share the common γ chain (γc, CD132), it is reasonable to speculate that GABA and propofol acting on GABA_A_ receptors may inhibit γc signaling. Further investigations are required to clarify the mechanism of propofol-mediated action in T cells.

This study has several limitations. First, the effect of propofol was evaluated in a mouse model of immunization and anesthesia, which does not always replicate human responses. Propofol was administered once daily by bolus intravenous injection for five consecutive days, which is different from the dosing regimens in clinical practice, where after induction of anesthesia, propofol is usually administered by continuous infusion lasting for several hours for the maintenance of anesthesia. When used for sedation of mechanically ventilated patients in the intensive care unit, propofol may be administered by continuous infusion for up to seven days. In this study, due to technical difficulties in continuous intravenous infusion in mice, repetitive bolus injection was employed. It remains unclear to what extent differences in dosing regimens influence the effect of propofol on the immune system. Second, intralipid was administered in equal doses to the lipid in propofol, but the rate of administration exceeded that recommended for humans [20], which may have affected the results. Third, the mechanisms by which the direct effect of propofol on T cells affects antibody production remains unclarified. Propofol-treated NP–KLH–immunized mice showed a reduction in both total and high-affinity NP-specific IgG1 titers and lacked an increase in the ratio of high-affinity to total NP-specific IgG1 in the secondary response, suggesting that affinity maturation may be affected by propofol treatment. In this regard, IL-7 signaling in T cells has been shown to influence B cell survival and memory B cell differentiation into antibody-producing cells [21].

In summary, our study provides evidence that repeated propofol treatment of mice inhibits T cell–dependent antibody production. The optimal interval between immunization and propofol anesthesia may be reconsidered to allow a sufficient humoral immune response to vaccines.

## Supplementary Information

Below is the link to the electronic supplementary material.

Supplementary file1 (PDF 4704 KB)

## Data Availability

The data that support the findings of this study are available from the corresponding authors (TH and HS) upon reasonable request.
