# Supplementary material for: Intravenous anesthetic propofol suppresses T cell–dependent antibody production in mice
Source: J Anesth. 2025 Jun 20;39(6):906–15. doi: 10.1007/s00540-025-03533-7 (PMC12647247; doi:10.1007/s00540-025-03533-7)
Supplement: Supplementary file 1 — Supplementary file1 (PDF 4704 KB) [file 540_2025_3533_MOESM1_ESM.pdf]

Journal of Anesthesia

Supplementary Materials for:

Intravenous anesthetic propofol suppresses T cell–dependent antibody production in mice

Susumu Hiraoka[1,2] • Hiroki Satooka[1] • Hirotoshi Kitagawa[2] • Takako Hirata[1]

✉ Takako Hirata

tahirata@belle.shiga-med.ac.jp

✉ Hiroki Satooka

hsatooka@belle.shiga-med.ac.jp

1 Department of Fundamental Biosciences, Shiga University of Medical Science, Otsu, Shiga 520-2192, Japan

2 Department of Anesthesia, Shiga University of Medical Science, Otsu, Shiga 520-2192, Japan

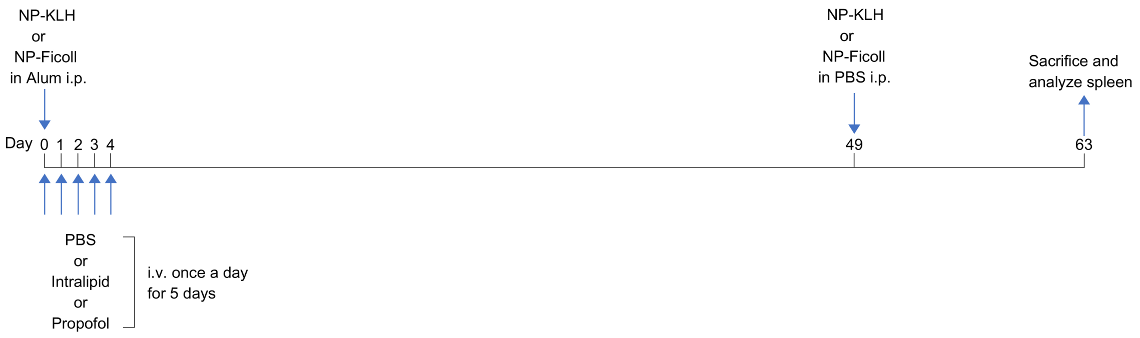

Supplementary Fig. 1 Schematic representation of experimental protocol. Mice were immunized with NP–KLH or NP–Ficoll in alum on day 0 and re-immunized with the same antigen in PBS on day 49. The mice were treated with propofol, intralipid, or PBS for five consecutive days from day 0 to day 4.

a

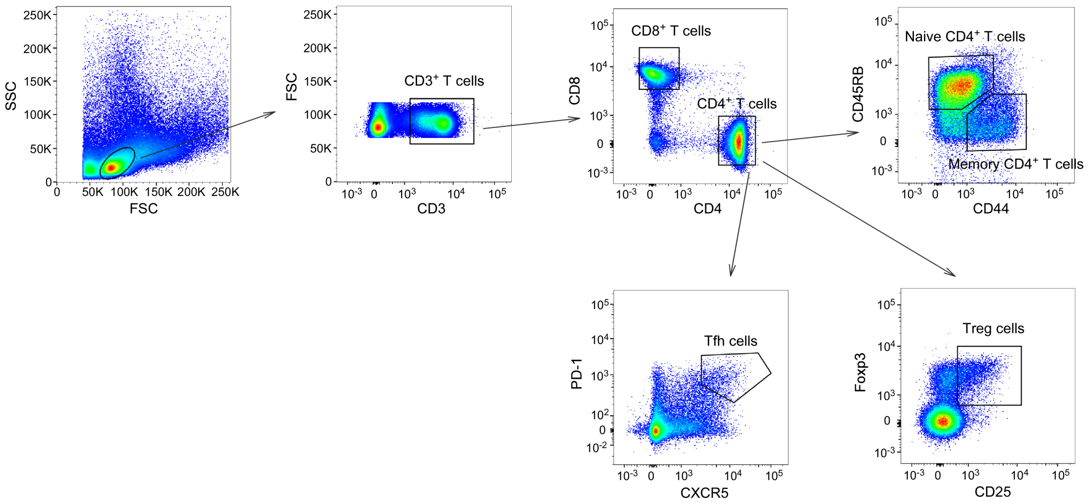

b

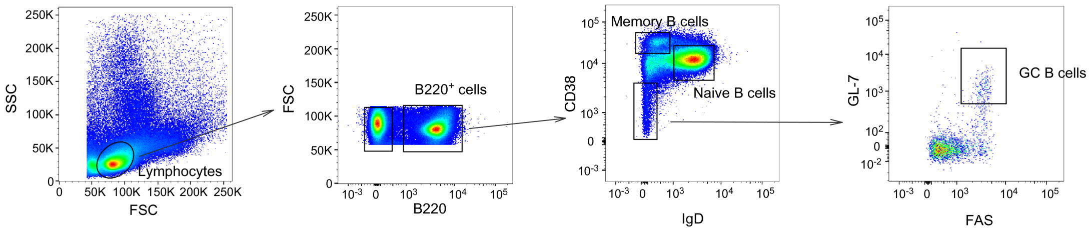

Supplementary Fig. 2 Gating strategies used for flow cytometry. **a** Gating strategies for T cell subsets from mouse spleens. The gates for T cells ($CD3^+$), $CD4^+$ T cells ($CD3^+CD4^+$), $CD8^+$ T cells ($CD3^+CD8^+$), naive $CD4^+$ T cells ($CD3^+CD4^+CD44^{lo}CD45RB^{hi}$), memory $CD4^+$ T cells ($CD3^+CD4^+CD44^{hi}CD45RB^{lo}$), Treg cells ($CD3^+CD4^+CD25^+Foxp3^+$), and Tfh cells ($CD3^+CD4^+CXCR5^+PD\text{-}1^+$) are shown. **b** Gating strategies for B cell subsets from mouse spleens. The gates for B cells ($B220^+$), naive B cells ($B220^+IgD^+CD38^+$), memory B cells ($B220^+IgD^-CD38^{hi}$), and GC B cells ($B220^+IgD^-CD38^-FAS^+GL\text{-}7^+$) are shown.

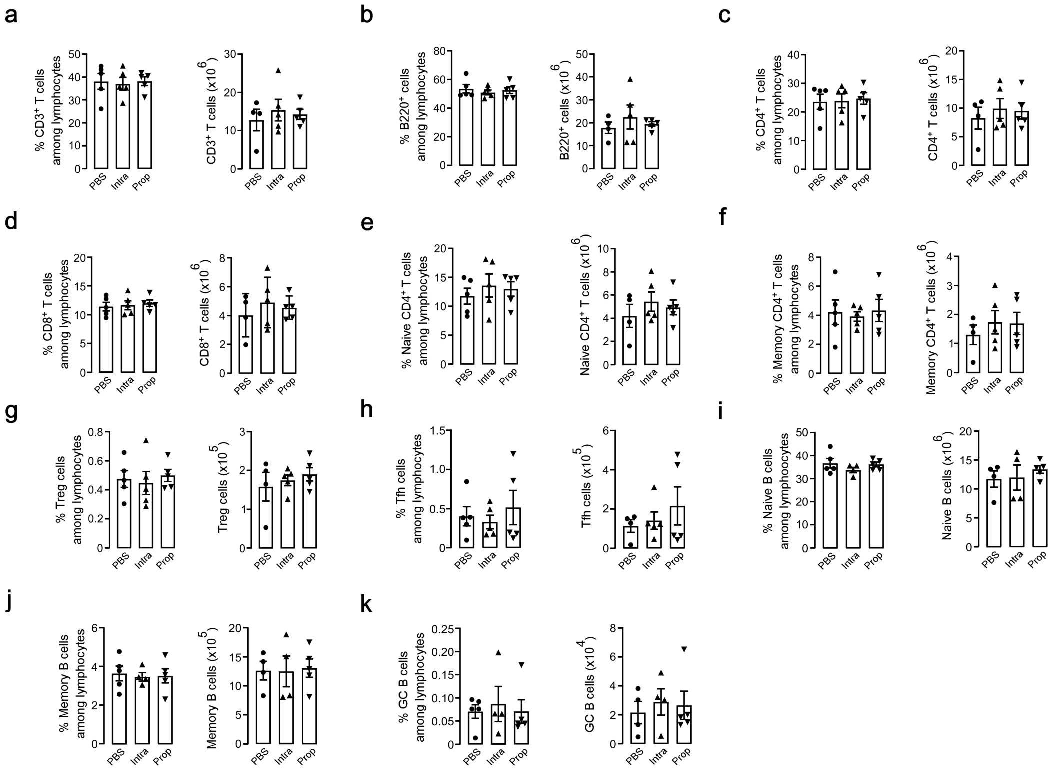

Supplementary Fig. 3 Effects of propofol on T and B cell subsets in the spleen of mice immunized with T cell–independent antigen. The percentage and number of T cells (**a**), B cells (**b**), $CD4^+$ T cells (**c**), $CD8^+$ T cells (**d**), naive $CD4^+$ T cells (**e**), memory $CD4^+$ T cells (**f**), Treg cells (**g**), Tfh cells (**h**), naive B cells (**i**), memory B cells (**j**), and GC B cells (**k**) in the spleen isolated on day 63 from NP–Ficoll–immunized mice treated with PBS, intralipid, or propofol. Data are presented as mean ± SEM (n = 5 mice per group).

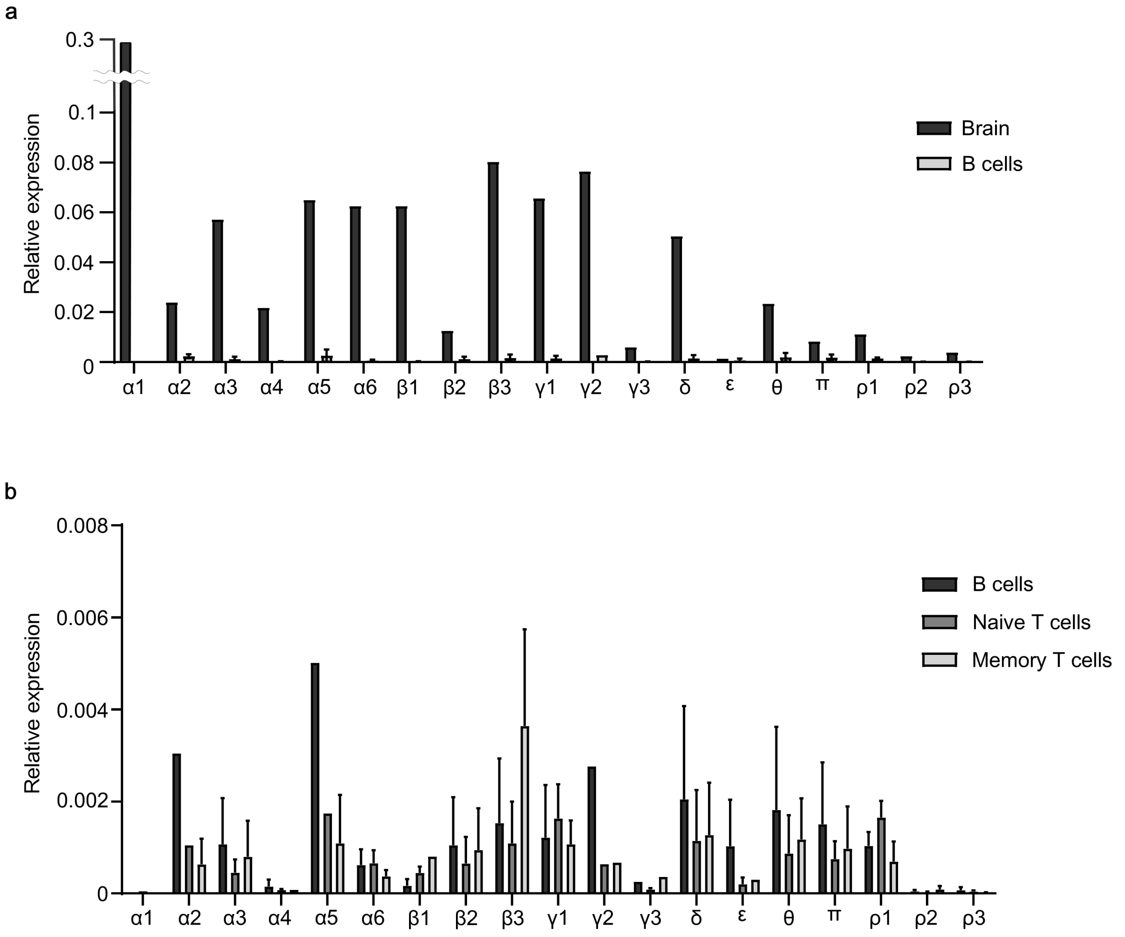

Supplementary Fig. 4 GABA$_A$ receptor subunit expression in the brain and B and T cells. Relative expression of GABA$_A$ receptor subunit mRNAs in the brain and B cells (**a**) and in T cell subsets in comparison with B cells (**b**). The mRNA levels were assessed by quantitative PCR and normalized to β-actin levels. Total RNA from mouse brain tissue, B cells, naive and memory CD4$^+$ T cells was extracted using TRIzol Reagent (Invitrogen). RNA was reverse transcribed using ReverTra Ace qPCR RT Master Mix with gDNA Remover (Toyobo). Quantitative PCR was performed using KOD SYBR qPCR Mix (Toyobo) and a LightCycler 480 instrument (Roche). The primer pairs were described previously [Ref. 9 of the main paper].

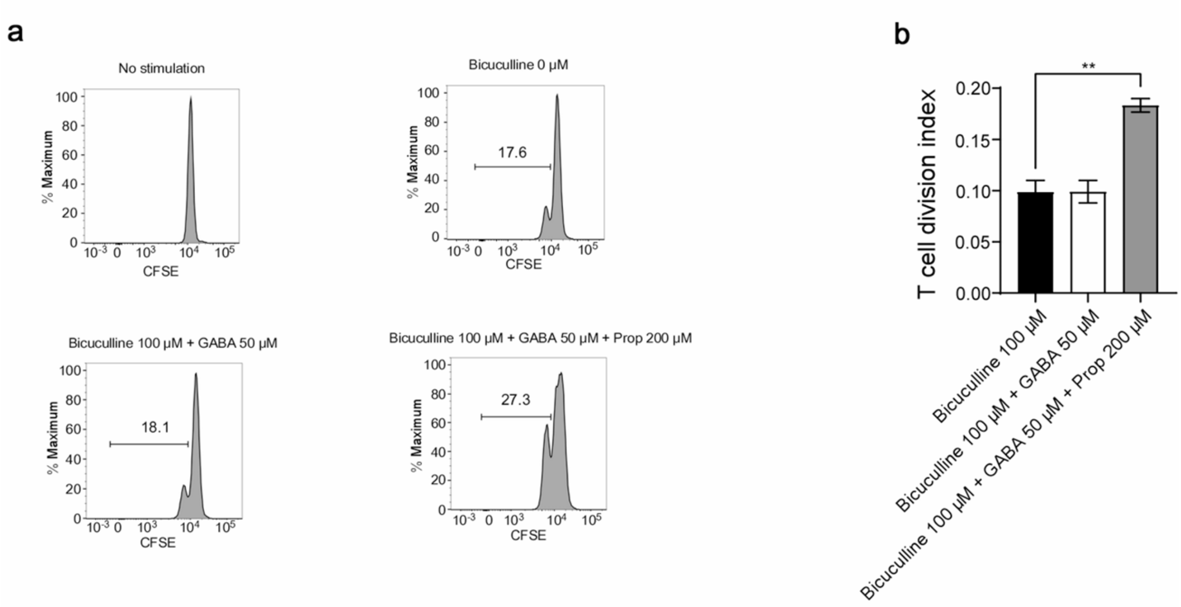

Supplementary Fig. 5 T cell proliferation in the presence of bicuculline, GABA, and propofol. Representative histograms (**a**) and T cell division index (**b**) of CFSE-labeled naive $CD4^+$ T cells stimulated with anti-CD3, anti-CD28, and IL-2 in the presence of 100 μM bicuculline (TCI) with or without GABA and propofol. Data are presented as mean ± SEM (n = 3 wells per group). $^{**}p < 0.01$.

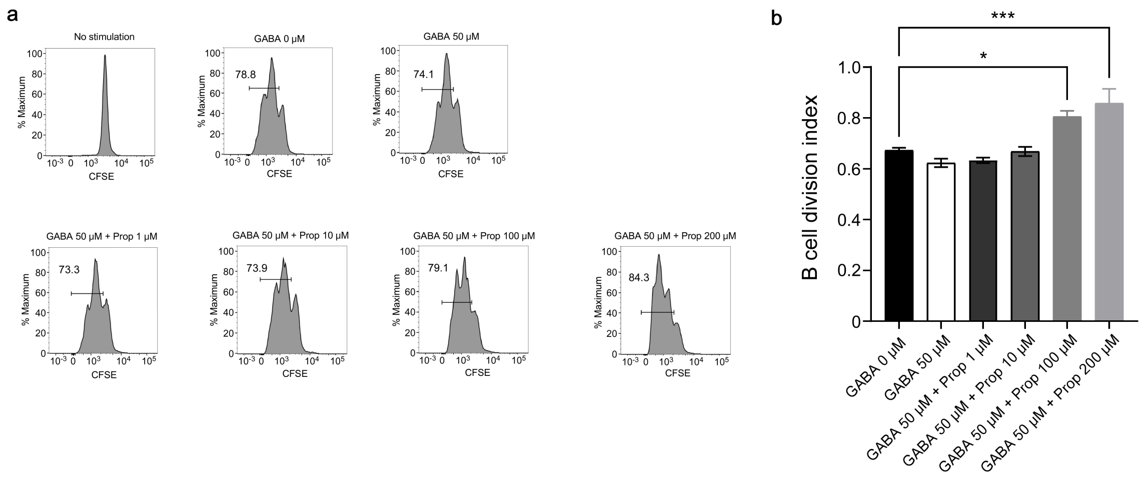

Supplementary Fig. 6 B cell proliferation in the presence of GABA and propofol. Representative histograms (**a**) and B cell division index (**b**) of CFSE-labeled B cells stimulated with LPS and anti-IgM with or without GABA and propofol. B cells were isolated from splenocytes using EasySep Mouse B Cell Isolation Kit (Stemcell Technologies), stained with CFSE, and stimulated with 5 μg/ml LPS and anti-IgM with or without GABA and propofol for 72 h. Data are presented as mean ± SEM (n = 4 wells per group) and represent three independent experiments. *$p < 0.05$, ***$p < 0.001$.
